# Interventional Radiological Treatment of Liver Tumours

**DOI:** 10.1038/sj.bjc.6605102

**Published:** 2009-06-09

**Authors:** T Meyer

**Affiliations:** 1 UCL Cancer Institute, Paul O′Gorman Building, University College London, 72 Huntley Street, London WC1E 6BT, UK

Therapeutic radiological interventions have now become well established in the treatment algorithms and clinical guidelines for hepatocellular cancer, and are increasingly being used in colorectal and neuroendocrine tumours metastatic to the liver. As the evidence base for these interventions is growing and new technologies arriving, this book presents a timely review of the current modalities available. The opening chapter gives us a broad review of the management of primary liver cancer and colorectal liver metastasis, and is helpful in placing the subsequent chapters in context. The new and important breakthroughs in systemic therapies, such as sorafenib for hepatocellular cancer, are also briefly reviewed, raising the exciting prospect for their use in combination with local therapies. Subsequent chapters cover commonly used modalities, including percutaneous ethanol injection (PEI), transarterial chemoembolisation (TACE) and radiofrequency ablation (RFA). Practical and technical advice is provided for each intervention, along with a review of complications and evidence for efficacy. Although PEI and TACE are dealt with in single chapters, a full three chapters are devoted to RFA covering the science, the practice and giving advice about how to set up a radiofrequency service. There are also chapters covering less familiar techniques, such as cryoablation and high-intensity focused ultrasound (HIFU), which were particularly helpful, but the omission of a chapter on selective internal radiation therapy (SIRT) was disappointing given the current level of interest in this area both for hepatocellular cancer, and for colorectal and neuroendocrine tumours. By contrast, the two chapters on the histopathology and surgery of liver tumours, although well written, appear slightly out of place in a book that is otherwise focused on radiological intervention.

What is clear from these thorough reviews is that there are few well-powered clinical trials to define the optimal intervention or technique. The majority of studies referred to are often retrospective, single-arm, single-centre studies with a limited number of small prospective randomised trials. Consequently the text is, on occasion, biased towards the local practice of the author, while the lack of evidence for some aspects of practice is acknowledged. For example TACE, which has been used for the treatment of hepatocellular carcinoma (HCC) worldwide for decades, is not a standardised procedure and is performed differently in every institution. Questions about the use and type of chemotherapy, the use of lipiodol, the best embolic particle and the schedule remain unanswered, making an authoritative guidance difficult. The emergence of a standardised protocol, perhaps with drug-eluting beads (DEBs), may help to overcome the heterogeneity of practice, providing clinical trials demonstrate superior safety and efficacy. As the number of therapeutic options increase, the need to carefully define the role of each modality becomes more important. As Lencioni *et al* point out, this has been achieved to a great extent for PEI and TACE based on the findings from three randomised trials discussed in their chapter. From these studies the case for RFA for tumours over 2 cm seems convincing in terms of efficacy. However, given the cost of RFA and the fact that HCC is most common in developing countries, PEI remains an attractive option for early tumours. It is not yet clear where HIFU and cryotherapy will fit into the therapeutic algorithm. Like RFA, HIFU requires a general anaesthetic, but is considerably more time consuming and expensive.

The book has been edited by two eminent radiologists and is most clearly aimed at a radiological audience. As such it will be invaluable to trainees entering hepatobiliary radiology, but will also provide a useful review of the landscape for those already in the field. However, writing as a hepatobiliary oncologist, I found the book both accessible and informative and hope that it would be of interest to other members of the hepatobiliary multi-disciplinary team.

